# Clinical Audit of Acute Oxygen Therapy: Enhancing Patient Care at Dammar Teaching Hospital

**DOI:** 10.7759/cureus.92790

**Published:** 2025-09-20

**Authors:** Mohand Tag Elsser Mohammed Albadwy, Salma Abdalla Elshaikh, Ali Ahmed, Faris Jamalaldeen Mohammed Hamed, Amjad Ahmed, Mozaffar Sirelkhatim Elzain Abdalla, Amer Rababah, Marwa Abdelrazig Eljack Ibrahim, Mazin Mamoun Badawi Khalifa, Ahmed Shakir Ali Yousif, Mohammed Mohammednoor, Asim Faisal Awad Ibrahim, Islam Yahia Abdalrahman Yagoub, Ibrahim Elbashir, Mohamed Abdelbasit, Muaz Abdullah Ahmed Ali, Tasneem Sulieman M Tahameed, Hanaa Ahmed Khalifa Elamin, Mustafa Mohamed, Mohammed Sid Ahmed

**Affiliations:** 1 Surgery, National University, Khartoum, SDN; 2 Internal Medicine, Ministry of Health and Prevention (MOHAP), Ajman, ARE; 3 Emergency Medicine, Prince Abdulaziz Bin Musaad Hospital, Arar, SAU; 4 General Practice, Al Neelain University, Khartoum, SDN; 5 Emergency Medicine, Withybush General Hospital, Haverfordwest, GBR; 6 Anaesthesiology, Prince Othman Digna Teaching Hospital, Port Sudan, SDN; 7 General Practice, Jabal Al-Zaitoon Hospital, Amman, JOR; 8 General Practice, Omdurman Islamic University, Khartoum, SDN; 9 Respiratory Medicine, The Grange University Hospital, Cwmbran, GBR; 10 Urology, Gezira Hospital for Renal Diseases and Surgery, Wad Medani, SDN; 11 Medicine, University of Khartoum, Khartoum, SDN; 12 Colorectal Surgery, Beaumont Hospital, Dublin, IRL; 13 Emergency Medicine, University of Khartoum, Khartoum, SDN; 14 Emergency Medicine, King Khalid Civilian Hospital, Tabuk, SAU; 15 General Medicine, University of Sinnar, Khartoum, SDN; 16 General Medicine, National Ribat University, Khartoum, SDN; 17 Internal Medicine, Dongola Specialized Hospital, Dongola, SDN; 18 Internal Medicine, Prince Othman Digna Teaching Hospital, Port Sudan, SDN; 19 Internal Medicine, Dammar Teaching Hospital, Dammar, SDN

**Keywords:** british thoracic society (bts), clinical audit, guideline adherence, oxygen therapy, quality improvement

## Abstract

Background: Oxygen therapy is a critical component of acute care, yet inappropriate administration remains a frequent concern, particularly in resource-limited contexts such as Sudan. Challenges with guideline adherence, infrastructure, and staff training necessitate clinical audits for quality improvement.

Objective: The objective of the study is to assess compliance with oxygen therapy guidelines at Dammar Teaching Hospital and evaluate the impact of targeted interventions on optimizing acute oxygen delivery.

Method: The study is a prospective two-cycle clinical audit (Cycle 1: June-July 2024, n = 50; Cycle 2: December 2024, n = 40) against standards. Interventions included staff training and standardized checklists. Compliance was measured across nine parameters (device-specific flow rates, documentation, weaning, etc.).

Results: Compliance improved in several domains after the intervention. Correct device and flow use increased markedly for nasal cannula (50.0% to 92.3%) and non-rebreather masks (61.9% to 100.0%). Documentation also improved, with target SpO₂ recording in type 2 respiratory failure rising from 69.0% to 100.0% and routine SpO₂ monitoring from 50.0% to 82.1%. Immediate oxygen delivery for patients at risk of hypoxemia increased from 16.7% to 70.0%. However, compliance declined in oxygen weaning (76.2% to 45.0%) and timely discontinuation (78.6% to 47.5%).

Conclusion: Educational interventions and clinical tools were associated with improved adherence to guidelines in oxygen delivery and documentation. However, declines in weaning/discontinuation highlight persistent de-escalation challenges. Sustained progress may require iterative audits, protocol reinforcement, and integration into electronic health records, which could provide a scalable model for similar settings.

## Introduction

Oxygen therapy remains a cornerstone intervention in the management of acutely ill patients requiring respiratory support. Despite its widespread use, inappropriate oxygen administration, either over- or under-oxygenation, can lead to adverse outcomes, including increased morbidity and mortality [1,2]. Evidence from systematic reviews indicates that liberal oxygen supplementation is associated with increased mortality in acutely ill adults, emphasizing the risks of hyperoxia [3]. Conversely, hypoxemia remains a leading cause of preventable complications, particularly in emergency and critical care contexts [4].

Recent guidelines, such as those from the British Thoracic Society (BTS), emphasize the importance of targeted oxygen therapy tailored to individual patient needs. These guidelines advocate precise titration to achieve optimal oxygen saturation levels, generally within the range of 94%-98% for most patients and 88%-92% for those at risk of hypercapnic respiratory failure [5]. The BTS and related quality standards stress the use of formal oxygen prescriptions, documentation of target saturations, and ongoing monitoring as essential elements of safe practice [6].

Audits and established quality standards consistently demonstrate that adherence to oxygen therapy protocols is variable across healthcare systems. For example, international observational studies reveal substantial variation in oxygen delivery practices in ventilated patients, with many institutions exceeding conservative oxygen targets [7]. Such deviations highlight the necessity for continual evaluation of oxygen use practices within healthcare settings to ensure adherence to evidence-based protocols [4,6].

In resource-limited contexts such as sub-Saharan Africa, persistent challenges in oxygen provision, infrastructure, and staff training further complicate safe delivery [8,9]. A systematic review from Ethiopia showed that while healthcare workers recognized oxygen as life-saving, gaps in knowledge, practice, and confidence were common, largely due to inadequate training and inconsistent supply [10]. Similarly, during the COVID-19 pandemic, shortages of oxygen supplies across Africa exposed the fragility of oxygen systems and the urgent need for scalable solutions [8].

A recent study in Sudan highlighted significant gaps and barriers in healthcare workers’ understanding of oxygen therapy, with many clinicians citing a lack of structured protocols and insufficient continuing education as barriers to safe practice [11]. These findings underscore the importance of clinical audits and targeted educational interventions as tools to strengthen oxygen therapy practices in low-resource settings.

## Materials and methods

This quality improvement clinical audit was conducted at Dammar Teaching Hospital, a tertiary care center in Sudan, aimed at evaluating and improving the standards of acute oxygen therapy administered in both the emergency department and inpatient wards. A prospective cross-sectional observational design was utilized to capture real-world practice across two formal audit cycles. The study population included all patients receiving oxygen therapy during the audit periods, with 50 patients in the first cycle (June 20 to July 20, 2024) and 40 patients in the second cycle (December 1-20, 2024).

Audit standards and criteria

The audit criteria and standards were based on the BTS guideline for oxygen use in adults in healthcare and emergency settings (2017), which is freely accessible for clinical and academic use [9]. Oxygen therapy was administered according to the patient’s clinical condition and oxygen saturation levels, using a nasal cannula at flow rates of 2-6 L/min, a simple face mask at 6-10 L/min, and a non-rebreather mask at 10-15 L/min or higher. Oxygen saturation (SpO₂) was regularly monitored and recorded on observation charts. For patients with type 2 respiratory failure, the target oxygen saturation range was set between 88% and 92%, as documented by the treating physician. The target oxygen saturation was also clearly documented in patient notes. Oxygen flow rate was to be gradually reduced in stable patients while closely monitoring for any signs of desaturation. Oxygen therapy was discontinued promptly once patients maintained their target SpO₂ without supplemental oxygen. Immediate oxygen delivery was mandatory for patients at risk of developing hypoxemia. These standards provided measurable criteria for compliance and guided the audit interventions.

First cycle: baseline audit and problem identification

During the first audit cycle, patient charts and observation sheets were systematically reviewed for compliance with established oxygen therapy standards. Baseline data revealed significant gaps in care delivery and documentation. Only 50.0% of patients received oxygen via nasal cannula at the recommended flow rate of 2-6 L/min, while 59.5% received oxygen by a face mask at 6-10 L/min, and 61.9% by a non-rebreather mask at 10-15 L/min. Documentation of oxygen saturation on observation sheets was recorded in 50.0% of cases. The target oxygen saturation for patients with type 2 respiratory failure was documented in 69.0% of patients, while only 45.2% had the target SpO₂ recorded in the patient notes by the treating doctor. Oxygen flow rate reduction (weaning) in stable patients was achieved in 76.2% of cases, and 78.6% of patients were disconnected from oxygen once able to maintain saturation. However, immediate oxygen delivery for patients at risk of hypoxemia was critically low at 16.7%. Informal staff interviews and documentation reviews identified insufficient guideline awareness and a lack of standardized protocols or checklists as major barriers to optimal care.

Intervention: education and protocol implementation

Following a root cause analysis, targeted interventions were implemented. Educational sessions were arranged for emergency and ward staff to emphasize adherence to the BTS guidelines, including appropriate oxygen prescription, device selection and flow rates, documentation practices, and risks associated with improper oxygen therapy. Practical workshops and case discussions consolidated the learning points. Simultaneously, standardized checklists and visible clinical reminders were introduced in clinical areas to support real-time adherence, focusing on documentation of indications, target saturations, and proper oxygen weaning.

Second cycle: post-intervention audit and outcome assessment

The second audit cycle, conducted with the same methodology and criteria, showed marked improvements in most key parameters. Oxygen delivery via a nasal cannula and face mask at recommended flow rates increased to 92.3%, and use of the non-rebreather mask reached 100.0%. Recording of oxygen saturation on observation sheets improved to 82.1%, and documentation of target oxygen saturation for type 2 respiratory failure rose to 100.0%. The proportion of patients with target SpO₂ written in the patient notes by doctors increased to 79.5%. However, oxygen flow rate reduction in stable patients declined to 45.0%, and disconnection from oxygen once target saturation was maintained dropped to 47.5%, indicating the need for further emphasis on these aspects. Importantly, immediate oxygen delivery to patients at risk of hypoxemia improved significantly to 70.0%. These results confirm that audit-driven educational initiatives and real-time clinical prompts effectively enhance compliance with oxygen therapy standards, though ongoing attention is required to maintain progress across all areas.

Ethical considerations

This audit was approved by the hospital’s medical administration (IRB Number: 25/AU/0675). Patient confidentiality was maintained, and individual consent was not required as this was a registered quality improvement initiative.

## Results

A total of 90 patient records were reviewed across both cycles of the oxygen therapy audit at Dammar Teaching Hospital, with 50 patients in the first cycle and 40 in the second. Overall, a marked improvement was observed in adherence to oxygen delivery and documentation standards after educational interventions.

The most significant advance was seen in the proper use of nasal cannula at 2-6 L/min, which increased from 50% in the first cycle to 92.3% in the second-a 42.3 percentage point improvement. Use of face masks at 6-10 L/min improved substantially as well, from 59.5% to 92.3% (32.8% increase). Application of non-rebreather masks at the correct flow rate rose from 61.9% to 100% (an improvement of 38.1 percentage points).

Documentation also improved: recording oxygen saturation on observation sheets increased from 50% to 82.1% (32.1% gain), and documentation of the target oxygen saturation range for patients with type 2 respiratory failure (88%-92%) increased from 69% in the first cycle to 100% in the second (a 31% improvement). Similarly, the proportion of cases where the target SpO₂ was written in patient notes by the treating doctor rose from 45.2% to 79.5% (34.3 percentage point gain).

A particularly large improvement was seen in the immediate delivery of oxygen to patients at risk for hypoxemia, which increased from 16.7% to 70% (a 53.3 percentage point improvement). However, there was a decline in certain key areas: the rate at which oxygen flow was reduced in stable patients (weaning) dropped from 76.2% in the first cycle to 45% in the second (31.2 percentage point decrease), and the proportion of patients disconnected from oxygen once able to maintain saturation fell from 78.6% to 47.5% (a 31.1 percentage point reduction).

In summary, the educational interventions resulted in significant improvements in most aspects of oxygen therapy practice, especially regarding documentation and the use of appropriate oxygen delivery modalities. Nonetheless, the decline in weaning and timely discontinuation practices highlights areas that require further attention. Continued regular education, reinforcement of protocols, and periodic audits are recommended to sustain and further enhance adherence to best practice guidelines (Table 1).

**Table 1:** Compliance with British Thoracic Society (BTS) Oxygen Therapy Standards Before and After Intervention at Dammar Teaching Hospital The table compares compliance rates between the first audit cycle (June–July 2024, n = 50) and the second cycle (December 2024, n = 40). Parameters include oxygen delivery methods, documentation, weaning, and discontinuation practices. “Gap to 100% (first cycle)” indicates the percentage deficit from complete compliance during the initial cycle. Compliance with oxygen therapy standards across audit cycles. Parameters adapted from the BTS guideline for oxygen use in adults in healthcare and emergency settings [1].

Parameter	First cycle	Second cycle	Gap to 100% (first cycle)
Patient received oxygen by nasal cannula at 2–6 L/min	25 (50.0%)	37 (92.3%)	50.0%
Patient received oxygen by face mask at 6–10 L/min	30 (59.5%)	37 (92.3%)	40.5%
Patient received oxygen by non-rebreather mask at 10–15 L/min	31 (61.9%)	40 (100.0%)	38.1%
Oxygen saturation is recorded on the observation sheet	25 (50.0%)	33 (82.1%)	50.0%
Target oxygen saturation for type 2 respiratory failure (88%–92%)	35 (69.0%)	40 (100.0%)	31.0%
Target SpO₂ written in the patient notes by the treating doctor	23 (45.2%)	32 (79.5%)	54.8%
Oxygen flow rate reduced in a stable patient (weaning)	38 (76.2%)	18 (45.0%)	23.8%
Patient disconnected once able to maintain saturation	39 (78.6%)	19 (47.5%)	21.4%
Oxygen delivered immediately to patients at risk of hypoxemia	8 (16.7%)	28 (70.0%)	83.3%

## Discussion

This clinical audit at Dammar Teaching Hospital sought to evaluate and optimize the administration of acute oxygen therapy, highlighting the importance of evidence-based practices for patient safety and care quality. Over two audit cycles, marked improvements were observed across multiple parameters of oxygen delivery and documentation practices, reflecting both the efficacy of targeted interventions and ongoing challenges in guideline implementation.

Improvements were most notable in the precision of oxygen delivery: the proportion of patients receiving oxygen via nasal cannula at recommended flow rates increased from 50.0% to 92.3%, with similar gains for face mask use (92.3%) and non-rebreather mask use (100.0%). These trends suggest that focused education and staff engagement with protocols can rapidly translate into clinical practice improvements [1,2]. Similar improvements have been documented in international audits, where targeted education significantly narrowed the gap between guideline recommendations and bedside practice [7].

In parallel, crucial aspects of documentation improved: oxygen saturation was recorded on observation sheets in 82.1% of cases (up from 50.0%), and treating doctors were more proactive in specifying target saturations in the notes (rising from 45.2% to 79.5%). These changes are essential, particularly for patients with type 2 respiratory failure, where strict adherence to specified target SpO₂ ranges (88%-92%) is critical to prevent harm from hypoxemia or hyperoxia. The IOTA meta-analysis demonstrated that liberal oxygen use beyond target ranges was associated with increased mortality, reinforcing the importance of accurate prescription and monitoring [3]. Adherence in this group reached 100% after the intervention [5].

Despite these gains, some domains regressed between cycles. Oxygen weaning in stable patients fell from 76.2% to 45.0%, and prompt disconnection when adequate saturation was maintained decreased from 78.6% to 47.5%. This decline may be explained by greater clinician confidence in oxygen initiation than in de-escalation, concerns regarding patient deterioration, and the absence of standardized weaning protocols [12,13]. Additional contributing factors include staff turnover, workflow bottlenecks, and limited monitoring capacity, which are common in low-resource settings. Although immediate oxygen delivery to patients at risk of hypoxemia improved from 16.7% to 70.0%, this remains suboptimal, leaving a significant proportion of patients vulnerable to adverse outcomes [8-10].

Clinical implications

These findings highlight the value of structured re-education and audit cycles to embed guideline-based oxygen therapy practices sustainably [1-6]. Regular auditing has been shown to drive sustained behavior change, particularly when combined with feedback and point-of-care prompts [14]. Challenges such as staff turnover, documentation habits, and clinical workflow bottlenecks need continuous attention. To sustain improvements, future strategies should include integration of oxygen therapy protocols into electronic health records, ongoing staff training and simulation exercises, regular audit and feedback cycles, and promotion of interdisciplinary communication. Solutions may also include establishing standardized workflows and checklists to maintain consistent practice [5,6]. Furthermore, WHO and global pediatric data stress that reliable access to oxygen, combined with trained staff, could reduce preventable mortality from pneumonia and other acute illnesses, underscoring the global significance of local improvements [9,15].

Limitations

This audit’s limitations include its single-center design and relatively small sample size, which may limit generalizability. Observer bias, or the “audit effect,” is possible, as staff behavior may have changed due to awareness of monitoring. The accuracy and completeness of clinical documentation varied, potentially affecting data reliability. Follow-up was limited to short-term outcomes without assessment of long-term clinical impact on morbidity or mortality, which is a common constraint in audit-based studies [14]. Additionally, outcome data-including complications, mortality, and hospital stay, particularly in patients where weaning protocols were not appropriately followed-were not consistently available and thus could not be included. Future audit cycles should integrate these outcomes to more clearly assess the clinical consequences of suboptimal oxygen practices.

Future directions

To address remaining gaps, further interventions such as simulation training, point-of-care reminders, and leveraging health IT for compliance prompting are recommended. Wider multicenter studies could validate these findings and explore the role of institutional context in intervention success. Regional reviews suggest that combining simulation-based education with robust supply-chain interventions can significantly strengthen oxygen systems [10]. Long-term monitoring of patient-centered outcomes-including morbidity, mortality, and hospital stay duration-will be critical for demonstrating the clinical benefits of these practice improvements [3,7]. Future cycles should also assess the sustainability of improvements and benchmark findings against international audits to strengthen generalizability and applicability.

## Conclusions

Targeted educational interventions and protocol standardization at Dammar Teaching Hospital significantly improved acute oxygen therapy practices, evidenced by enhanced compliance with device-specific flow rates (e.g., nasal cannula: 50%→92.3%; non-rebreather mask: 61.9%→100%) and documentation (target SpO₂ for type 2 respiratory failure: 69%→100%). These gains mirror global evidence confirming the efficacy of structured training, clinical prompts, and audit cycles. However, critical regressions in oxygen weaning (76.2%→45.0%) and timely discontinuation (78.6%→47.5%) emerged, highlighting persistent de-escalation challenges. Sustained adherence requires iterative audit cycles, electronic health record integration, and focused reinforcement of weaning protocols. This framework offers a scalable model for optimizing oxygen safety in lower-resource settings.
